# Perturbation of copper homeostasis is instrumental in early developmental arrest of intraerythrocytic *Plasmodium falciparum*

**DOI:** 10.1186/1471-2180-14-167

**Published:** 2014-06-24

**Authors:** Hiroko Asahi, Mohammed Essa Marghany Tolba, Masanobu Tanabe, Sumio Sugano, Kazumi Abe, Fumihiko Kawamoto

**Affiliations:** 1 Department of Parasitology, National Institute of Infectious Diseases, 23-1 Toyama 1-chome, Shinjuku-ku, Tokyo 162-8640, Japan; 2 Department of Medical Genomics, Graduate School of Frontier Sciences, The University of Tokyo, 4-6-1 Shirokanedai, Minato-ku, Tokyo 108-8639, Japan; 3 Department of Parasitology, Faculty of Medicine, Assiut University, Assiut 71515, Egypt; 4 Department of Infectious Diseases, Keio University School of Medicine, 35 Shinanomachi, Shinjuku-ku, Tokyo 160-8582, Japan; 5 Institute of Tropical Disease, Campus C, Airlangga University, JL. Mulyorejo Surabaya 160115, Indonesia

**Keywords:** *Plasmodium falciparum*, Intraerythrocytic growth, Copper homeostasis, Copper-binding protein, Copper ion, Developmental arrest

## Abstract

**Background:**

Malaria continues to be a devastating disease. The elucidation of factors inducing asexual growth versus arrest of *Plasmodium falciparum* can provide information about the development of the parasite, and may help in the search for novel malaria medication. Based on information from genome-wide transcriptome profiling of different developmental stages of *P. falciparum*, we investigated the critical importance of copper homeostasis in the developmental succession of *P. falciparum* with regard to three aspects of copper function. These were:1) inhibition of copper-binding proteins, 2) copper-ion chelation, and 3) down-regulated expression of genes encoding copper-binding proteins associated with a specific growth-promoting factor.

**Results:**

Inhibition of copper-binding proteins with tetrathiomolybdate (TTM) caused cessation of growth of the parasite. TTM arrested the parasite irreversibly during the trophozoite to schizont stage progression. Target molecules for TTM may be present in *P. falciparum.* The involvement of copper ions in developmental arrest was also investigated by copper-ion chelating methods, which indicated a critical function of reduced copper ions (Cu^1+^) in the parasite during the early developmental stage. Copper ions, not only in the parasite but also in host cells, were targets of the chelators. Chelation of Cu^1+^caused blockage of trophozoite progression from the ring stage. Profound growth arrest was detected in parasites cultured in a chemically defined medium containing hexadecanoic acid alone as a growth-promoting factor. This developmental arrest was associated with down-regulated expression of genes encoding copper-binding proteins. *Cis*-9-octadecenoic acid completely prevented the down-regulation of gene expression and developmental arrest that were observed with the use of hexadecanoic acid.

**Conclusions:**

The critical importance of copper homeostasis in early developmental stages of *P. falciparum* was confirmed. Perturbation of copper homeostasis induced profound and early developmental arrest of *P. falciparum*. These findings should help to elucidate the mechanisms behind the development of *P. falciparum*, and may be applied in the development of effective antimalarial strategies.

## Background

Malaria continues to be a devastating disease, particularly in the tropics, with an estimated annual incidence worldwide of 90 million clinical cases. The annual mortality from malaria, which is caused largely by the protozoan *Plasmodium falciparum*, is estimated to be 627,000 worldwide [1]. A better understanding of antimalarial treatments and the biology of the parasite is therefore needed, to allow the development of new medications to combat resistance to conventional antimalarial drugs [2].

The *P. falciparum* parasite develops through three distinct stages within red blood cells (RBCs) during its cycle of approximately 48 h: the ring, trophozoite, and schizont stages [3]. However, the mechanisms responsible for the developmental succession are poorly understood. A complete understanding of the functional molecules involved in developmental succession/arrest may provide clues for future efforts in drug and vaccine development aimed at eradicating malaria.

In order to identify the factors that control intraerythrocytic development of *P. falciparum*, we have previously investigated growth-promoting substances in order to formulate a chemically defined culture medium (CDM) suitable for sustaining the complete development and intraerythrocytic growth of *P. falciparum*[4,5]. Further, we have compared genome-wide transcriptome responses among different developmental stages of *P. falciparum* cultured in various CDMs with different growth-promoting effects, and selected 26 transcripts that were expected to be associated with the suppression of schizogony. Of these, five transcripts were considered to be particularly closely associated with the blockage of trophozoite progression from the ring stage, because of profound differences in transcript levels between the ring and trophozoite stages. One is a putative copper channel (a putative Ctr copper transporter domain containing protein, PF3D7_1421900 at PlasmoDB [6]; XP_001348385 at the National Center for Biotechnology Information, NCBI). In addition, selective removal of Cu ions has been shown to inhibit completely the successive ring–trophozoite–schizont progression of *P. falciparum*[7].These results suggest the involvement of copper homeostasis in the early developmental stages of intraerythrocytic *P. falciparum*.

In the present study we investigated in detail the importance of copper homeostasis for the development of *P. falciparum*, with regard to three aspects of copper function: 1) inhibition of copper-binding proteins that regulate copper physiology and function by actively associating with copper ion(s), 2) copper-ion chelation, and 3) down-regulated expression of genes encoding copper-binding proteins, in association with arrested development of the parasite caused by a specific growth-promoting factor.

## Methods

### Parasites, cultures, and synchronization

Cultures of the FCR3/FMG (FCR3, Gambia) strain of *P. falciparum* were used in all experiments. The parasites were maintained using *in vitro* culture techniques. The culture medium was devoid of whole serum and consisted of basal medium (CRPMI) supplemented with 10% of a growth-promoting fraction derived from adult bovine plasma (GFS) (GF21; Wako Pure Chemical Industries, Osaka, Japan), as reported [8]. This complete medium is referred to as GFSRPMI. The CRPMI consisted of RPMI-1640 containing 2 mM glutamine, 25 mM 4-(2-hydroxylethyl)-piperazine ethanesulfonic acid, 24 mM sodium bicarbonate (Invitrogen Ltd., Carlsbad, CA, USA), 25 μg/ml gentamycin (Sigma-Aldrich Corp., St. Lowis, MO, USA) and 150 μM hypoxanthine (Sigma-Aldrich). Briefly, RBCs were preserved in Alsever’s solution [8] for 3–30 days, washed, dispensed into 24-well culture plates at a hematocrit of 2% (1 ml of suspension/well), and cultured in a humidified atmosphere of 5% CO_2_, 5% O_2_, and 90% N_2_ at 37°C. The parasitemia was adjusted to 0.1% (for subculture) or 0.3% (for growth tests) by adding uninfected RBCs, unless specified otherwise, and the hematocrit was adjusted to 2% by adding the appropriate volume of culture medium.

The CDMs consisted of CRPMI containing bovine serum albumin free of any non-esterified fatty acid (NEFA) at a final concentration of 3 mg/ml. This was supplemented further with NEFAs, individually or in combination. The following phospholipid supplements were also added: 15 μM 1,2-dioleoyl phosphatidic acid sodium salt, 130 μM 1,2-dioleoyl-sn-glycerol-3-phosphocholine, 25 μM 1,2-dioleoyl-sn-glycero-3-phosphoethanolamine, and 15 μM 1,2-dioleoyl-sn-glycero-3-phosphoserine, sodium salt. The CDMs included CDRPMI that was supplemented with both 60 μM hexadecanoic acid (C16:0) and 100 μM *cis*-9-octadecenoic acid (C18:1) as NEFAs and CDM-C16alone, which contained 160 μM C16:0 alone. All compounds were obtained from Sigma-Aldrich, unless specified otherwise. Dried lipid precipitates were prepared, added to the culture media, and sterilized to reconstitute the lipids, as described previously [4].

Cultures were synchronized at the ring stage by three successive exposures to 5% (w/v) D-sorbitol (Sigma-Aldrich) at 41- and 46-h intervals [9]. After the third sorbitol treatment, residual schizonts and cell debris were removed by isopycnic density centrifugation on 63% Percoll PLUS (GE Healthcare Bio-Sciences, Tokyo, Japan). Parasites at the ring stage (adjusted to 5.0% parasitemia, unless specified otherwise) were maintained for growth experiments in synchronized cultures.

### Evaluation of growth inhibition

Growth inhibition was measured by adding graded concentrations of inhibitors or chelators, including ammonium tetrathiomolybdate (TTM, Sigma-Aldrich), 2,9-dimethyl-1,10-phenanthroline, hydrochloride, monohydrate (Neocuproine, Tokyo Chemical Industry, Co., Tokyo, Japan), *bis*(cyclohexanone) oxaldihydrazone (Cuprizone, Merck Japan, Ltd., Tokyo, Japan), and 2,9-dimethyl-4,7-diphenyl-1,10-phenanthrolinedisulfonic acid, disodium salt (BCS, Sigma-Aldrich). The IC_50_ values (the concentration required to inhibit the growth of the parasite by 50% compared with inhibitor-free controls) were extrapolated from the concentration–response curves.

In all the experiments, the culture wells were run in triplicate or quadruplicate. All experiments were repeated two to four times.

### Assessment of parasite growth

Samples were taken at indicated times after inoculation. Thin smears were made and stained with Giemsa. Parasitemia was determined by examining more than 10,000 infected RBCs (PfRBCs)/uninfected RBCs. The growth rate was estimated by dividing the parasitemia of the test sample after the indicated incubation period by the initial parasitemia.

### RNA preparation

*P. falciparum* was isolated from PfRBCs (160 μl packed PfRBCs at 5% parasitemia) at the end of the incubation period (28 h) by lysing infected cells, followed by centrifugation (1750 *g*, at 4°C for 10 min). The isolated parasites were preserved in RNAprotect Cell Reagent (QIAGEN GmbH, Hilden, Germany) to protect the nucleic acids of the parasites from degradation. Total RNA was harvested from the parasites using the RNase plus Micro kit (QIAGEN), following the manufacturer’s protocol. The concentration of harvested RNA was confirmed using NanoDrop ND-100 (Thermo Fisher Scientific Inc., Yokohama, Japan).

### Quantitative real-time PCR (qRT-PCR)

Analysis of gene expression (transcripts) for the target genes was performed by qRT-PCR on *P. falciparum* cultured in various media, and also for the housekeeping gene glycerol-3-phosphate dehydrogenase (GPDH, XM_001350529.2 at NCBI). Diluted RNA samples were subjected to the Applied Biosystems StepOnePlus Real-Time PCR System, using a Power SYBR Green RNA-to-C_T_™ 1-Step kit according to the protocol given in the handbook. The final PCR volume was 20 μl in 96-well plate format, containing 10 μl 2 × Power SYBR Green PCR Master Mix, 0.16 μl Reverse Transcriptase Mix, and 2 μl of 1 μM of each primer. The cycling conditions were 48°C for 30 min, 95°C for 10 min, followed by 40 cycles of 95°C for 15 s, 60°C for 1 min. The One-step RT-PCRs were carried out under identical conditions in triplicate, and assay controls with no template and with no reverse transcriptase were also tested to exclude the possibility of contamination and to discriminate between primer dimers and small amplicons with low melting temperatures. The difference in threshold cycle (C_T_) values (∆C_T_) between the C_T_ values of the target gene and those of the GPDH gene were taken as a marker of gene expression levels in the same samples. Real-time results are expressed as a quotient of the levels of transcripts. Stringent specificity controls included melting curve analysis for each target mRNA amplification.

Primer sets that exhibited the lowest C_T_ values were selected from 5–10 primer sets for each mRNA. The primers employed were: (1) a putative copper channel (XM_001348349.1 at NCBI), forward 5′-TGCCTGACCTTCACTTTCGATT-3′ and reverse 5′-CATAGGTAACATAACTCCATCGTCA-3′; (2) a copper transporter (XM_001348507.1 at NCBI), forward 5′-CTATGCCAATGTCCTTTCAGC-3′ and reverse 5′-CTTCCGTTTTTGGCAAGG-3′; (3) a putative cytochrome C oxidase copper chaperone (putative COX17; XM_001347500.1 at NCBI), forward 5′-CACGAATGAAGCAAATAAAGGAG-3′ and reverse 5′-CTGCTCTTCCCCCAATTTAAC-3′; (4) a copper-transporting ATPase (Cu^2+^-transporting ATPase; XM_001351887.1 at NCBI), forward 5′-ACCCGAGGTTTTTGAACTAATC-3′ and reverse 5′-AACCTTCTCTAAGGGCAACG-3′; (5) a transcription factor with AP2 domains (AP2-O; XM_001348075.1 at NCBI), forward 5′-AGCCAAGATACTGTTATTGTTGATG-3′ and reverse 5′-TCCCCTCTTTCCTTTCACTC-3′; (6) a guanylyl cyclase (GCalpha; XM_001348029.1 at NCBI), forward 5′-TGGCTTGTACCTGTGATGTTG-3′ and reverse 5′-TCATCGCTATGTCATTTGCAC-3′; (7) GPDH, forward 5′-TAGTGCTTTGTCAGGGGCTAAC-3′ and reverse 5′-CCATCACAAAATCCGCAAG-3′.

### Statistical analysis

The significance of the differences between means was evaluated using multifactorial analysis of variance. All calculations were performed using GraphPad PRISM 5 (GraphPad Software, Inc., San Diego, CA, USA). The *P* value for significance was 0.05, and all pairwise comparisons were made post hoc with Bonferroni’s test. Error bars were added to the y-axes on the graphs to indicate the standard deviation for each point.

## Results

### Effect of TTM on growth of *P. falciparum*

**Figure 1:**
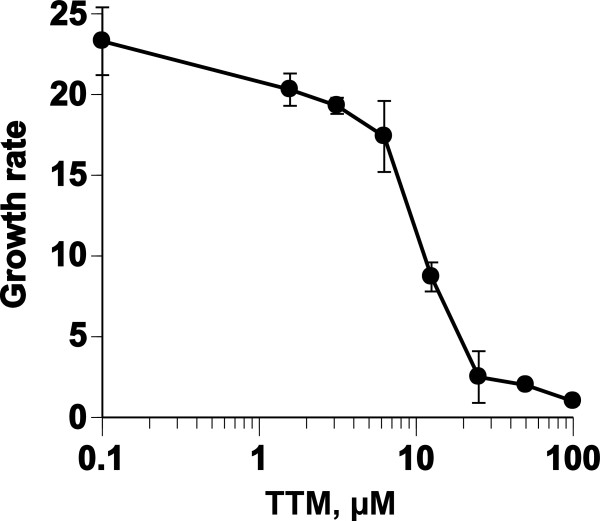
**Growth-arresting effect of TTM on asynchronous *P. falciparum *parasites.** Parasites were cultured in GFSRPMI for 95 h in the presence of graded concentrations of TTM. The IC_50_ of TTM is 12.3 ± 0.1 μM.

TTM inhibits copper-binding proteins through formation of a metal cluster, rather than by direct chelation of copper ions [10]. The effect of TTM on the growth of asynchronous *P. falciparum* was examined by adding graded concentrations of TTM to the GFSRPMI culture. The addition of TTM caused cessation of growth in cultures of the parasite (Figure 1, IC_50_ = 12.3 ± 0.1 μM).

**Figure 2:**
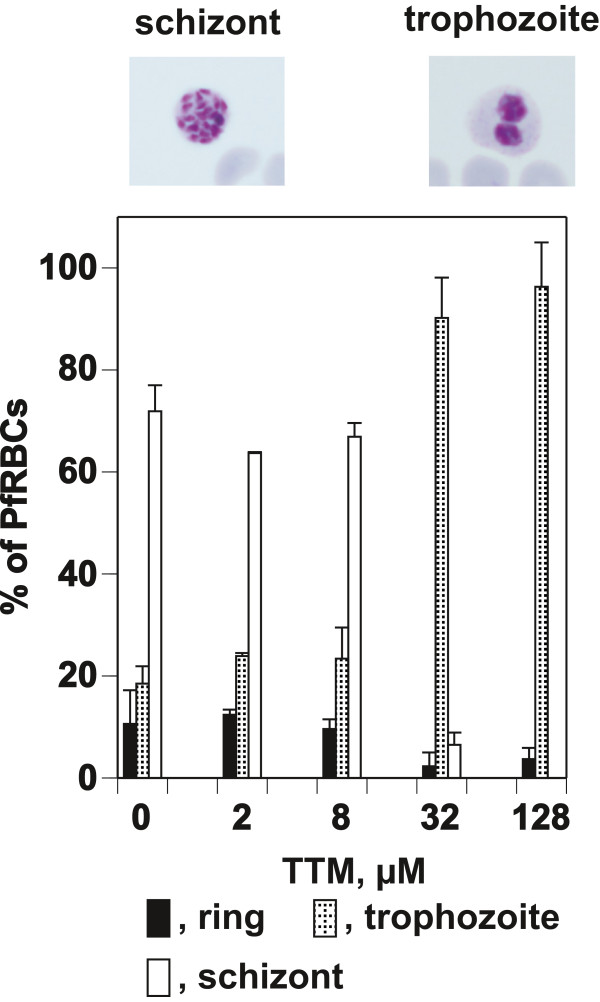
**Effect of TTM on growth of synchronized *P. falciparum *parasites.** Synchronized parasites at the ring stage were cultured in GFSRPMI for 28 h in the presence of graded concentrations of TTM. Each developmental stage was counted after Giemsa staining. Levels of parasitemia were 5.33 ± 0.15 (0 μM TTM), 4.93 ± 0.12 (2 μM), 3.75 ± 0.24 (8 μM), 3.69 ± 0.26 (32 μM), and 3.23 ± 0.26 (128 μM). The morphology of the trophozoites observed in the presence of higher concentrations of TTM and the schizonts in the absence of TTM is shown above graph.

To determine the effect of TTM on the progression of *P. falciparum* parasites through the cell cycle, graded concentrations of TTM were added to GFSRPMI cultures of parasites synchronized at the ring stage. These cultures were allowed to develop for 28 h, sufficient time for growth to the schizont stage. The TTM arrested the parasite during the trophozoite–schizont stage progression. All stages of the parasite were observed at lower concentrations (2 and 8 μM) at various levels, but only trophozoites were observed at higher concentrations (32 and 128 μM) (Figure 2).

**Figure 3:**
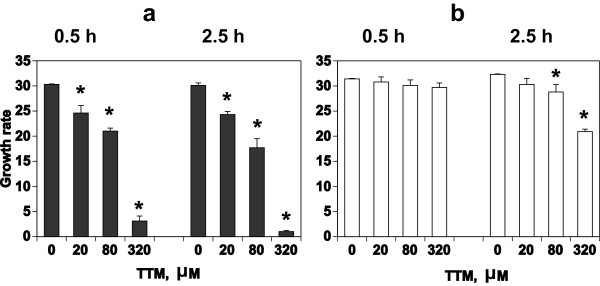
**Growth of *P. falciparum *co-cultured with PfRBCs and RBCs that were pretreated separately with TTM.** Synchronized PfRBCs at the ring stage and RBCs were treated with graded concentrations of TTM for 0.5 h or 2.5 h at room temperature. After washing, both treated PfRBCs and RBCs were mixed (pretreated PfRBCs plus non-treated RBCs **(a)** or non-treated PfRBCs plus pretreated RBCs **(b)**) at a ratio of more than 10 times RBCs to PfRBCs and cultured in GFSRPMI for 95 h; (*) indicates a significant difference versus no treatment with TTM (0).

To determine the location of target copper-binding proteins that are involved in the growth arrest of the parasite, and to study the role of TTM in the interaction between parasites and RBCs, an approach was applied in which PfRBCs and RBCs were treated separately and then mixed. PfRBCs at higher than 5% parasitemia were treated with TTM for 0.5 h and 2.5 h at room temperature. After washing, PfRBCs and uninfected RBCs were mixed at ratios of more than 1:10, and cultured in GFSRPMI for 95 h (two cycles). *P. falciparum* that had been pretreated with TTM showed profound growth arrest, even after a short period of treatment such as 0.5 h (Figure 3a). The inhibition was dose dependent. However, treatment of uninfected RBCs caused growth arrest to a lesser extent, and only at higher concentrations of TTM (80 μM and 320 μM) and with longer periods of treatment (2.5 h) (Figure 3b). Similar results were shown with cultures in CDRPMI. These results implied that, although TTM affects copper-binding proteins in RBCs, the target molecule(s) for TTM that are involved in the growth arrest of the parasite may occur predominantly in *P. falciparum*. Furthermore, TTM may react irreversibly with the copper-binding proteins of the parasite, or the parasites may take up TTM that remains even after washing, from RBCs.

### Effect of copper chelators on growth of *P. falciparum*

**Figure 4:**
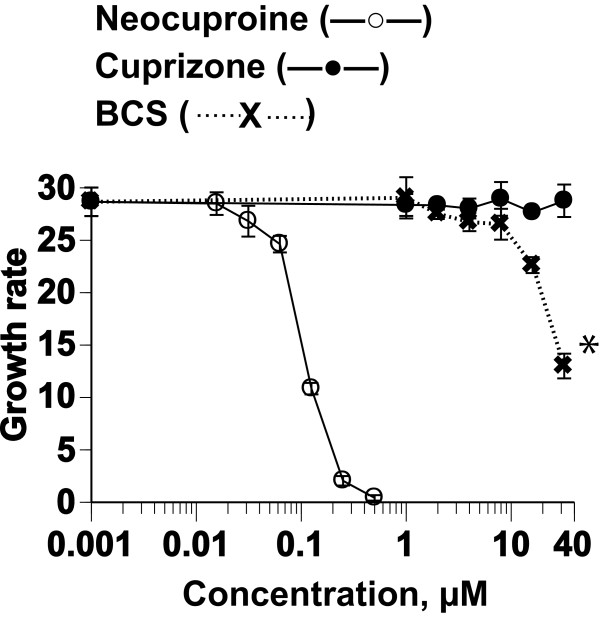
**Effect of various copper chelators on growth of asynchronous *P. falciparum *parasites.** Parasites were cultured in CDRPMI for 95 h in the presence of graded concentrations of the copper chelators Neocuproine, Cuprizone, and BCS; (*) indicates a significant difference versus no BCS. The IC_50_ of Neocuproine is 0.13 ± 0.06 μM.

The effect of copper ions on the growth of *P. falciparum* was examined by adding copper chelators to the CDRPMI culture. The chelators employed included two intracellular chelators, Neocuproine and Cuprizone, and one extracellular chelator, BCS. The addition of Neocuproine caused cessation of growth in asynchronous cultures of the parasite (IC_50_ = 0.13 ± 0.06 μM), whereas Cuprizone and BCS had no visible effect on the growth of the parasite, except at the higher concentration of BCS (32 μM) (Figure 4). The IC_50_ was similar to that of cultures in GFSRPMI (IC_50_ = 0.10 ± 0.01 μM [7]). Neocuproine selectively chelates reduced copper ions (Cu^1+^) by bidentate ligation and can diffuse through the cell membrane, while BCS, which chelates Cu^1+^ and the oxidized copper ion Cu^2+^, cannot cross the membrane. The cell membrane is permeable to Cuprizone, which chelates Cu^2+^[11]. The finding that only Neocuproine inhibited development of the parasite effectively indicates that Cu^1+^, but not Cu^2+^, is involved in the mechanisms responsible for the growth arrest of the parasite.

**Figure 5:**
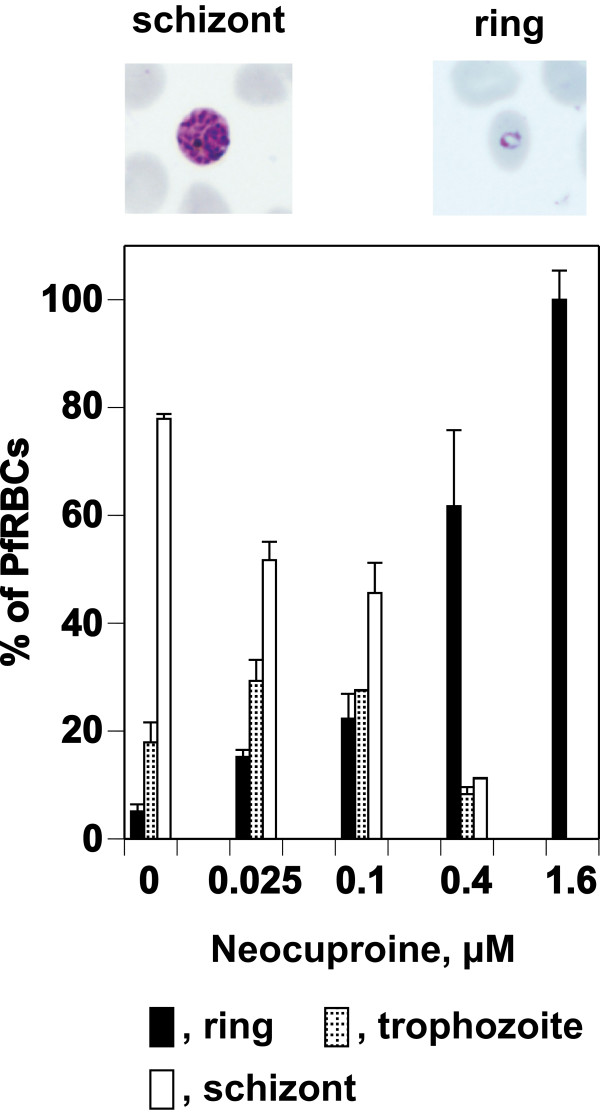
**Effect of Neocuproine on growth of synchronized *P. falciparum *parasites.** Synchronized parasites at the ring stage were cultured in CDRPMI for 28 h in the presence of graded concentrations of Neocuproine. Each developmental stage was counted after Giemsa staining. Levels of parasitemia were 7.60 ± 0.17 (0 μM Neocuproine), 7.44 ± 0.06 (0.025 μM), 7.63 ± 0.08 (0.1 μM), 7.08 ± 0.59 (0.4 μM), and 6.84 ± 0.37 (1.6 μM). The morphology of the rings observed in the presence of higher concentrations of Neocuproine and the schizonts in the absence of Neocuproine is shown above graph.

The effect of Cu^1+^ on the development of synchronized *P. falciparum* parasites at the ring stage was tested further by adding graded concentrations of Neocuproine to CDRPMI cultures, followed by culture for 28 h. Neocuproine arrested parasites during the ring–trophozoite–schizont stage progression, in a concentration-dependent manner similar to the results for cultures in GFSRPMI [7]. All stages of the parasite were observed at lower concentrations (0.025, 0.1, and 0.4 μM) at various levels, but only rings were observed at higher concentrations (1.6 μM) (Figure 5).

**Figure 6:**
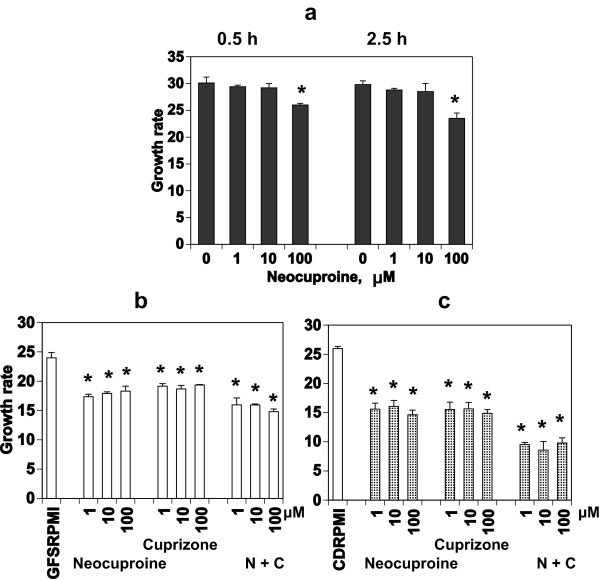
**Growth of *P. falciparum *co-cultured with PfRBCs and RBCs that were pretreated separately with the chelators.** Synchronized PfRBCs at the ring stage and RBCs were treated with graded concentrations of Neocuproine and/or Cuprizone for 0.5 h or 2.5 h at room temperature. After washing, both treated RBCs and PfRBCs were mixed (pretreated PfRBCs plus non-treated RBCs **(a)** or non-treated PfRBCs plus pretreated RBCs **(b, c)**) at a ratio of more than 10 times RBCs to PfRBCs, and cultured in GFSRPMI (b) or CDRPMI (a, c) for 95 h. RBCs were pretreated for 2.5 h (b, c); (*) indicates a significant difference versus no treatment with Neocuproine and/or Cuprizone. (N + C) indicates the mixture of Neocuproine and Cuprizone (1:1).

To determine the location of the target copper ions that are involved in the growth arrest of the parasite, and of the copper chelators involved in the interaction between the parasite and RBCs, an approach was applied in which PfRBCs and RBCs were treated separately and then mixed, similar to the experiments with TTM. PfRBCs at higher than 5% parasitemia were treated with the copper chelator Neocuproine, for 0.5 h and 2.5 h at room temperature. After washing, PfRBCs and uninfected RBCs were mixed at ratios of more than 1:10, and cultured for 95 h. Growth of *P. falciparum* that was pretreated with Neocuproine and co-cultured with uninfected and non-treated RBCs was arrested only with the high concentration of Neocuproine (100 μM), and to a very low extent (Figure 6a). This is in contrast to the results for *P. falciparum* cultured in the presence of Neocuproine throughout the culture period (48 h to 96 h) (Figure 4). Pretreatment of uninfected RBCs with two copper chelators, Neocuproine (for Cu^1+^) and Cuprizone (for Cu^2+^), individually or in combination, caused partial growth arrest of the parasite, and the effect was independent of the concentrations tested (Figure 6b). To avoid a possible effect of intrinsic copper ions in the surrounding culture medium, GFSRPMI, tests were also performed in CDRPMI, and showed similar results (Figure 6c). These results implied that chelation of Cu^1+^ ions of the parasite by Neocuproine may be reversible, or that Cu ions (Cu^1+^ and Cu^2+^) may be replenished by RBCs, because removal of Cu ions from RBCs caused growth arrest (Figure 6b,c).

### Arrested development of the parasite with CDM-16alone, and profoundly down-regulated expression of copper-binding proteins

The CDMs formulated for the development of *P. falciparum* contain specific NEFAs and phospholipids with specific fatty acid moieties. The effectiveness of the different NEFAs in sustaining the development of *P. falciparum* varies markedly, depending on their type, total amount, and combination, and the result ranges from complete development to growth arrest at the ring stage. The most effective combination of NEFAs has been found to be C18:1 and C16:0 [4,5].

**Figure 7:**
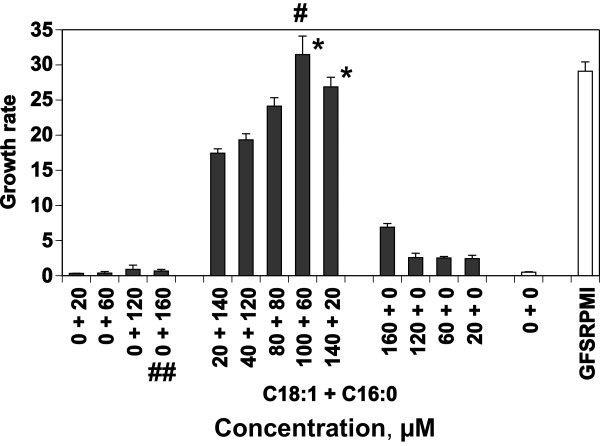
**Growth of asynchronous *P. falciparum *cultured for 95 h in the presence of NEFAs.** The two NEFAs, C18:1 and C16:0, were added to CDM, alone or in combination, at various concentrations and ratios. GFSRPMI was tested for comparison; (*) indicates no significant difference compared with GFSRPMI. (^#^) CDRPMI, (^##^) CDM-C16alone.

*P. falciparum* was cultured asynchronously with different concentrations and ratios of two NEFAs (C18:1 and C16:0), individually or in combination, in the presence of phospholipids. The mixtures of NEFAs, but not individual C16:0 or C18:1, sustained parasite growth (Figure 7). The NEFAs required pairing at different ratios: the maximum effect was obtained with 100 μM C18:1 plus 60 μM C16:0. This culture medium represents CDRPMI, and the growth rate was comparable to that in GFSRPMI. These experiments also showed that profound growth arrest of the parasite occurred in CDM enriched with either C16:0 or C18:1 (Figure 7).

**Figure 8:**
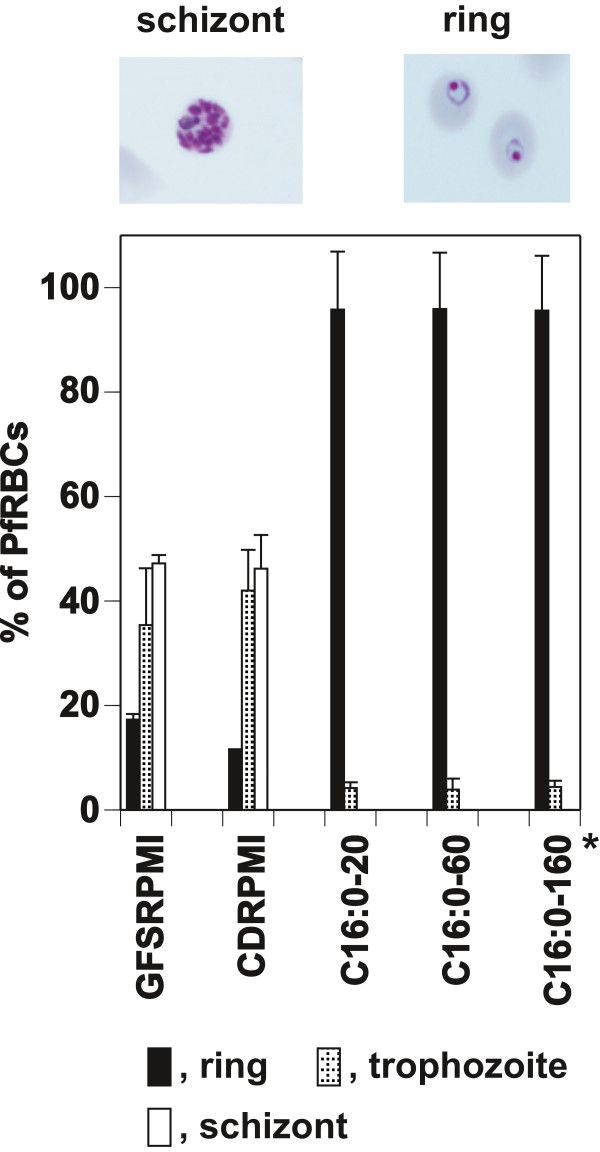
**Modification of *P. falciparum *development in CDMs containing C16:0 only as a NEFA growth factor.** Synchronized parasites at the ring stage were cultured in CDM containing graded concentrations of C16:0 (C16:0–20, 20 μM; C16:0–60, 60 μM; C16:0–160, 160 μM) for 28 h. Each developmental stage was counted after Giemsa staining. Levels of parasitemia were 5.27 ± 0.08 (GFSRPMI), 5.27 ± 0.34 (CDRPMI), 3.61 ± 0.30 (C16:0–20), 3.69 ± 0.60 (C16:0–60), and 3.67 ± (C16:0–160); (*) indicates CDM-C16alone. The morphology of the rings observed in the presence of C16:0 and the schizonts in GFSRPMI and CDRPMI is shown.

The profound growth arrest of *P. falciparum* was investigated further by culturing parasites synchronized at the ring stage in CDM containing different concentrations of C16:0, which was added individually, for 28 h. Suppression of schizogony, particularly the progression of the parasite to the trophozoite stage following the ring stage, was detected in CDM containing C16:0 alone as the NEFA growth factor, regardless of a wide range of concentrations (Figure 8). On the other hand, all stages of parasites cultured in CDRPMI had comparable development to those cultured in GFSRPMI (Figure 8). This implies that C18:1 protected the parasite completely from C16:0-induced growth arrest.

Although profound growth arrest was detected in *P. falciparum* cultured in CDM containing C18:1 alone for a longer period (95 h), all stages of the parasite cultured for 28 h had comparable development to those cultured in CDRPMI and GFSRPMI. However the majority of merozoites were incomplete, resulting in a low growth rate during the longer culture period (Figure 7). Thus, the growth arrest associated with CDM containing C18:1 alone did not involve suppression of schizogony.

**Figure 9:**
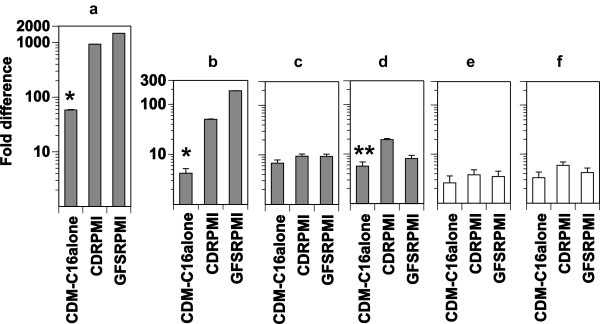
**Change in transcript levels.** Putative copper channel **(a)**, copper transporter **(b)**, putative COX17 **(c)**, copper-transporting ATPase **(d)**, AP2-O **(e)**, and GCalpha **(f)** of *P. falciparum* cultured for 28 h in CDM-C16alone, CDRPMI, and GFSRPMI were analyzed by qRT-PCR. Fold difference was calculated using ∆C_T_ (2^n^: n = ∆C_T_); (*) indicates significant difference versus CDRPMI and GFSRPMI and (**) versus CDRPMI.

Developmental arrest of *P. falciparum* was detected at the early stage in CDM-C16alone, similar to that with CDRPMI and GFSRPMI in the presence of Neocuproine and TTM, which cause perturbation of copper homeostasis. We have predicted previously, using genome-wide transcriptome profiling, five transcripts associated with the blockage of trophozoite progression from the ring stage [7], of which one transcript was a putative copper channel (PF3D7_1421900 at PlasmoDB [6]). This suggests a critical function of copper ions and copper-binding proteins in the early developmental arrest of the parasite, in agreement with the results with Neocuproine and TTM. Genes encoding proteins that are involved in the copper pathway and trafficking in various microbes have been identified in *P. falciparum.* These proteins include: 1) a putative copper channel (XP_001348385 at NCBI), 2) a copper transporter (XP_001348543.1 at NCBI), 3) a putative COX17 (XP_001347536 at NCBI), and 4) a copper-transporting ATPase (XP_001351923 at NCBI). The expression of the genes of these proteins was investigated further by qRT-PCR on cultures grown in CDM-C16alone. In *P. falciparum* cultured in CDM-C16alone, levels of transcripts of the putative copper channel and the copper transporter were profoundly decreased, and those of the copper-transporting ATPase to a lesser extent (Figure 9) in comparison with those in CDRPMI and GFSRPMI. The transcript level of the putative COX17 was not significantly different among the media, similar to those of AP2-O and GCalpha, which served as controls for transcript levels of non-copper related proteins (Figure 9).These results may indicate that down-regulation of the putative copper channel, the copper transporter, and the copper-transporting ATPase affects copper pathways and trafficking, and eventually causes the perturbation of copper homeostasis and growth arrest of the parasite. This implies also that the mono-unsaturated NEFA, C18:1, completely prevented the down-regulation of the gene expression observed with C16:0.

## Discussion

Copper ions are essential trace nutrients for all higher plants and animals at extremely low concentrations. They play an extensive role in living organisms, from microbes to plants and animals, by regulating the activities of several critical copper-binding proteins such as cytochrome c oxidase, Cu/Zn superoxide dismutase, dopamine β-hydroxylase, prion protein, tyrosinase, X-linked inhibitor of apoptosis protein, lysyl oxidase, metallothionein, ceruloplasmin, and other proteins [12,13]. Particularly in relation to microbes, copper ions are critical participants in the mitochondrial respiratory reaction and in energy generation, regulation of iron acquisition, oxygen transport, the cellular stress response, antioxidant defense, and several other important processes. The yeast *Saccharomyces cerevisiae* provides an accessible model for eukaryotic copper transport. Uptake of the Cu^2+^ ion by yeast cells is accompanied by reduction of Cu^2+^ to Cu^1+^ by a metalloreductase in the plasma membrane. Subsequent transport of the Cu^1+^ ion across the plasma membrane is carried out by a copper transporter (Ctr). Within the cell, Cu^1+^ ions are bound to the copper chaperones Atx1, Cox17, and CCS for specific delivery to the Golgi complex, mitochondria, and Cu/Zn superoxide dismutase, respectively [14]. Although there is no comprehensive understanding of copper metabolism and function in *P. falciparum*, the proteins involved in copper pathways and trafficking have been identified in *Plasmodium* spp. These include a putative copper channel, a copper transporter, a putative COX17, and a copper-transporting ATPase [6,15,16].

TTM has been known inhibit copper-binding proteins that regulate copper physiology through formation of a sulfur-bridged copper–molybdenum cluster, rather than by direct chelation of copper ions [10]. In the current study, TTM caused profound cessation of the growth of *P. falciparum*; this arrest resulted from inhibition of schizogony of the parasite. In contrast, treatment of uninfected RBCs with higher concentrations of TTM caused only slight growth arrest. Thus, the target molecule(s) of TTM may be present predominantly in the parasite, although the molecule(s) involved in the growth arrest of the parasite remain to be determined. Also, the possibility that the excess TTM affects, directly or indirectly, various proteins that do not bind to copper, and thus causes developmental arrest of the parasite, remains to be elucidated.

Chelation with Neocuproine, which selectively removes Cu^1+^[11], inhibited the successive ring–trophozoite–schizont progression of *P. falciparum* effectively at extremely low concentration; blockage of trophozoite progression from the ring stage was shown at higher concentrations. In contrast, the growth of *P. falciparum* pretreated with Neocuproine was arrested only to a very small extent, even when treated with much higher concentrations. This is quite different from the profound developmental arrest of *P. falciparum* maintained in the presence of Neocuproine throughout the culture period. We surmise that either the binding of Neocuproine may be reversible or copper ions may be replenished by host cells. RBCs contain copper at levels as high as a mean value of 18 μM, although most of the copper present in RBCs is bound to the enzyme superoxide dismutase [17,18].

Developmental arrest of *P. falciparum*, similar to that in CDRPMI and GFSRPMI in the presence of Neocuproine and TTM, was detected in the parasite cultured in CDM-C16alone. We have demonstrated previously, using genome-wide transcriptome profiling and various CDMs, profound down-regulation of the putative copper channel in parasites cultured in CDM-C16alone. This was associated with the blockage of trophozoite progression from the ring stage of the parasite. In the current study, the expression of genes encoding copper-binding proteins of *P. falciparum* was investigated, in detail, with cultures in CDM-C16alone, CDRPMI, and GFSRPMI. Transcript levels of not only a putative copper channel, which has previously been detected by genome-wide transcriptome profiling [7], but also a copper transporter were profoundly decreased during the arrested development of the parasite at the ring stage in CDM-C16alone. The severe down-regulation of copper-binding proteins of the parasite cultured in CDM-C16alone is considered to affect copper pathways and trafficking; this maybe involved in the perturbation of copper homeostasis and developmental arrest of the parasite, similar to the growth arrest seen with TTM and Neocuproine. However, the additional involvement of other proteins, such as merozoite surface protein 2, a putative DEAD/DEAH box RNA helicase, a serine repeat antigen 3, and a palmitoyl acyltransferase, which have been demonstrated to be associated with developmental arrest of the early stage of the parasite cultured in CDM-C16alone [7], is not excluded.

In addition to their basal functions, such as acting as important intermediates in lipid biosynthesis, there is evidence that various NEFAs are involved in numerous biological processes, including activation of protein kinases and cell proliferation, differentiation, and death [19-21]. NEFAs also affect numerous cellular systems and functions, including regulation of gene expression, ion-channel functions, and membrane fusion [22-24]. Saturated NEFAs such as C16:0 have been reported to cause a significant increase in mitochondrial reactive oxygen species, mitochondrial DNA damage, mitochondrial dysfunction, induction of Jun-N-terminal kinase, apoptosis, and inhibition of insulin signaling in skeletal muscle cells. In this study, we detected, for the first time, a profound down-regulation of the transcripts of copper-binding proteins when the parasites were cultured in CDM-C16alone, which contains C16:0. In addition, developmental arrest of the parasite at the ring/trophozoite stage occurred in tandem with the profound decrease in transcript levels. C18:1 (oleic acid) has been reported to prevent the mitochondrial dysfunction and apoptosis induced by C16:0 (palmitic acid) [25]. Similarly, *P. falciparum* cultured in CDRPMI containing both C18:1 and C16:0 showed similar growth to the parasite in GFSRPMI, which implies that C18:1 protected the parasite from the developmental arrest induced by C16:0 and the decrease in transcript levels. The mechanisms responsible for the profound down-regulation of copper-binding proteins in the parasite associated with C16:0 remain to be elucidated.

## Conclusions

The critical importance of copper homeostasis in early developmental stages of *P. falciparum* was demonstrated. Perturbation of copper homeostasis with an inhibitor of copper-binding proteins and a Cu^1+^ chelator induced profound early developmental arrest of *P. falciparum*. Down-regulation of copper-binding proteins also caused severe developmental arrest. These findings may provide clues to an effective antimalarial strategy. Further clarification of the target molecules of TTM, the factor that reduces Cu^2+^ to Cu^1+^, and the parasite factors that interact at the molecular level with NEFAs should help to elucidate the mechanisms behind the development of *P. falciparum*.

## Abbreviations

AP2-O: Transcription factor with AP2 domains; BCS: 2,9-dimethyl-4,7-diphenyl-1,10-phenanthrolinedisulfonic acid, disodium salt; C_T_: Threshold cycle, ∆C_T_, the difference in C_T_ values; C16:0: Hexadecanoic acid; C18:1: *cis*-9-octadecenoic acid; CDM: Chemically defined culture medium; CDM-C16alone: CDM containing 160 μM C16:0; CDRPMI: CDM containing 60 μM C16:0 and 100 μM C18:1; COX17: Cytochrome C oxidase copper chaperone; CRPMI: Basal medium; GCalpha: Guanylyl cyclase; GFS: A growth-promoting fraction derived from adult bovine plasma; GFSRPMI: Complete medium containing GFS; GPDH: Glycerol-3-phosphate dehydrogenase; IC_50_: The concentration required to inhibit growth of the parasite by 50% compared with inhibitor-free controls; NCBI: The National Center for Biotechnology Information; NEFA: Non-esterified-fatty acid; PfRBC: RBC infected with *P. falciparum*; qRT-PCR: Quantitative real-time PCR; RBC: Red blood cell; TTM: Ammonium tetrathiomolybdate.

## Competing interest

The authors declare that they have no competing interests.

## Authors’ contributions

HA and MEMT conceived and designed the study. HA, MEMT, MT, KA, and FK performed parasite culture and the experiments, and analyzed the data. HA and MEMT coordinated the study. SS contributed to the interpretation of the results (PCR). All authors read and approved the final manuscript.
